# NASQAR: a web-based platform for high-throughput sequencing data analysis and visualization

**DOI:** 10.1186/s12859-020-03577-4

**Published:** 2020-06-29

**Authors:** Ayman Yousif, Nizar Drou, Jillian Rowe, Mohammed Khalfan, Kristin C. Gunsalus

**Affiliations:** 1 NYU Abu Dhabi Center for Genomics & Systems Biology, Division of Biological Sciences, Abu Dhabi, United Arab Emirates; 2 Center for Genomics & Systems Biology, Department of Biology, New York University, New York, 10003 United States

**Keywords:** Transcriptomics, Graphical user interface, Interactive visualization, Exploratory data analysis

## Abstract

**Background:**

As high-throughput sequencing applications continue to evolve, the rapid growth in quantity and variety of sequence-based data calls for the development of new software libraries and tools for data analysis and visualization. Often, effective use of these tools requires computational skills beyond those of many researchers. To ease this computational barrier, we have created a dynamic web-based platform, NASQAR (Nucleic Acid SeQuence Analysis Resource).

**Results:**

NASQAR offers a collection of custom and publicly available open-source web applications that make extensive use of a variety of R packages to provide interactive data analysis and visualization. The platform is publicly accessible at http://nasqar.abudhabi.nyu.edu/. Open-source code is on GitHub at https://github.com/nasqar/NASQAR, and the system is also available as a Docker image at https://hub.docker.com/r/aymanm/nasqarall. NASQAR is a collaboration between the core bioinformatics teams of the NYU Abu Dhabi and NYU New York Centers for Genomics and Systems Biology.

**Conclusions:**

NASQAR empowers non-programming experts with a versatile and intuitive toolbox to easily and efficiently explore, analyze, and visualize their Transcriptomics data interactively. Popular tools for a variety of applications are currently available, including Transcriptome Data Preprocessing, RNA-seq Analysis (including Single-cell RNA-seq), Metagenomics, and Gene Enrichment.

## Background

Genomic data has experienced tremendous growth in recent years due to the rapid advancement of Next Generation Sequencing (NGS) technologies [1, 2]. Common applications include transcriptome profiling; de novo genome sequencing; metagenomics; and mapping of genomic variation, transcription factor binding sites, chromatin modifications, chromatin accessibility, and 3D chromatin conformation. Single-cell versions of these (e.g. [3]) and newer methods — such as spatial transcriptomics (e.g. [4]), CRISPR-based screens (e.g. [5]), and multi-modal profiling (simultaneous quantification of proteins and mRNAs, e.g. [6]) — are rapidly proliferating as new technical innovations come on the scene (e.g. [7, 8]). As the volume of data and diversity of applications continue to grow, so does the number of software libraries and tools for the analysis and visualization of these datasets. Many of the available tools for genomic data analysis require computational experience and lack a graphical user interface (GUI), making them inaccessible to many researchers whose work depends on them. Some of the common challenges include:

- Knowledge and experience in various programming/scripting languages (R, Python, shell, etc.)
- Data munging: pre-processing and reformatting for use with specific tools
- Limited computational resources (cpu, memory, and disk storage)
- Installation of software packages and dependencies. Many required tasks can be time consuming and tedious due to issues such as satisfying software or hardware requirements and resolving software dependencies. In one study [9], almost half (49%) of the published omics software tools that were randomly surveyed were found to be “difficult to install”. Moreover, the rapid churn of operating system updates and hardware configurations contributes to the gradual decline of a tool’s impact, usability, and lifetime.
- Software tools developed by researchers within academia are usually less "user-friendly", due to either a lack of development resources or a lack of expertise in best practices for software engineering, such as cross-platform compatibility and user interface design [9]. For example, many available R GUI based tools, while featuring very useful and diverse functionality, lack simple error handling and/or informative feedback. This can render the application unmanageable if users cannot easily identify and remedy the causes of such errors.

NASQAR (Nucleic Acid SeQuence Analysis Resource) is a web-based platform that wraps popular high-level analysis and visualization tools in an intuitive and appealing interface. This platform addresses the above challenges by offering the following:

- Utilization of software and interface design best practices to craft user-friendly and intuitive tools that are based on commonly used analysis packages. This is important in order to lower the entry barrier to standard bioinformatics analysis and visualization workflows, thus providing greater independence for researchers with little or no programming experience. The platform may be used for QC, exploratory analysis, or production of publication-ready data files (such as normalized counts data) and figures (PCA plots, heatmaps, dendograms, UMAP/t-SNE etc.)
- A scalable virtualization architecture that is relatively simple to deploy on a personal computer, an organization’s private/public web servers, or on the cloud (AWS, Microsoft Azure, Google Cloud, etc.). Virtualization allows for the abstraction of software and operating system dependencies, thus alleviating difficulties in installation for end users. The scalable design is advantageous when deploying the platform online for multiple concurrent users, either for public use or internal use within a research facility. It uses open-source packages, which is particularly desirable for academic research institutions.
- Modular design of analysis categories. By decoupling data preprocessing, RNA-seq analysis, and gene enrichment applications from each other, users can leverage these functions independently, thus allowing a greater versatility of analysis steps than fully integrated workflows.

The NASQAR platform provides a highly accessible, scalable, and user-friendly framework for versatile data analysis, comprising a consolidated toolbox of publicly available open-source applications (curated and vetted for good value and design) and custom applications developed in-house. While many useful web-based bioinformatics applications are now available, most focus exclusively on one type of analysis or application (e.g. bulk or single-cell RNA-seq, metagenomics, etc.) A few examples – some of which are included in NASQAR – include START [10], DEApp [11], TCC-GUI [12], Shiny-seq [13], GENAVi, is-CellR [14], and Shaman [15]. Fully integrated end-to-end analysis workflows such as GENAVi [16] employ a variety of R packages and/or other tools to streamline consecutive sequence analysis tasks (e.g. from preprocessing all the way to gene enrichment). While often desirable, this approach also restricts the end-user from performing just one of the implemented functions (such as gene enrichment), which is particularly useful for datasets generated independently using other tools or by external collaborators. NASQAR takes a different approach, aiming instead to empower non-programming experts with a “Swiss army knife” to perform a variety of sequence analysis tasks on their own. These may be accessed either as independent units or sequentially, with convenient interfaces to commonly used R data analysis packages and functions. This flexible framework offers a model resource for the community that can be extended to a broader range of applications through further development and collaboration.

## Implementation

**Fig. 1:**
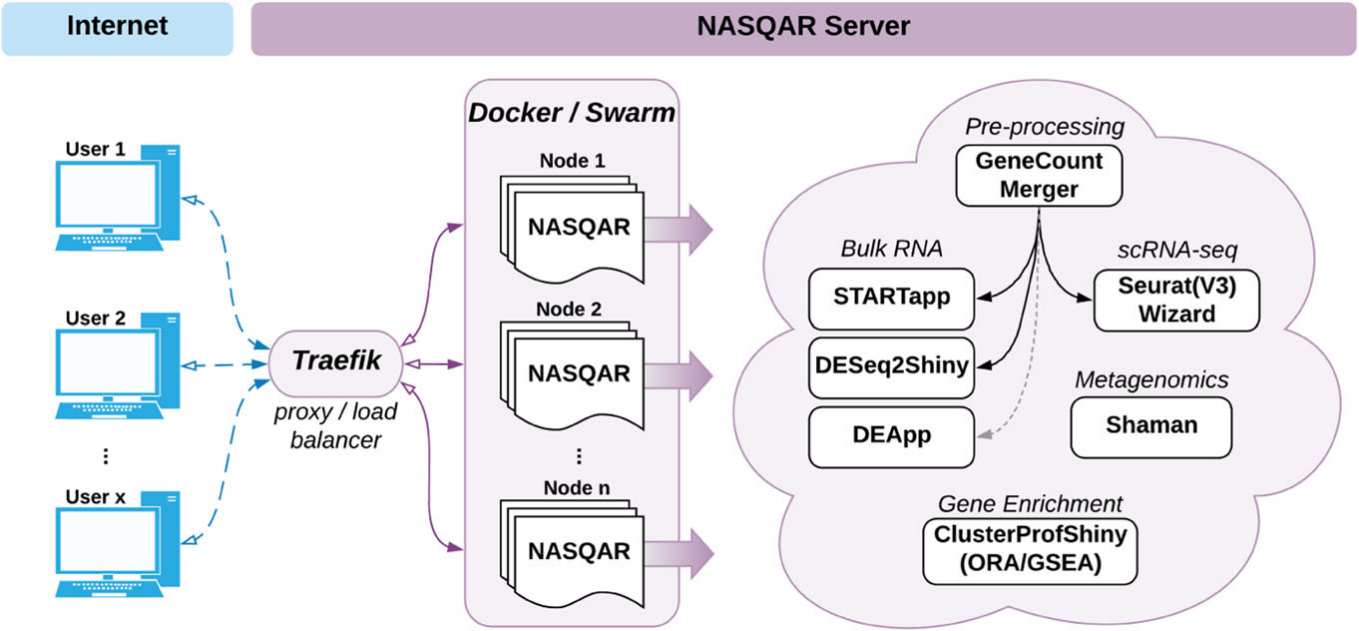
NASQAR platform architecture. A cluster of virtual machines at NYU Abu Dhabi serves NASQAR applications to multiple concurrent users. Applications are containerized and managed on the cluster using Docker and Swarm, while Traefik load-balances requests among available server nodes. Functionality includes merging gene counts, conversion of gene IDs to gene names, analysis of differential mRNA expression, metagenomics analysis, and functional enrichment analysis. Applications for bulk expression analysis include DESeq2, limma, and EdgeR. Single-cell RNAseq analysis with Seurat Wizards is built on top of the Seurat R package and includes options for filtering, normalization, dimensionality reduction (PCA), clustering, and UMAP/t-SNE. Enrichment analysis includes applications for Gene Set Enrichment Analysis (GSEA) and Over-representation Analysis (ORA) built using the clusterProfiler R package

The architecture of the NASQAR web platform is illustrated in Fig. 1. NASQAR has been deployed on a cluster of virtual machines and is publicly accessible at http://nasqar.abudhabi.nyu.edu/. Docker [17] and Swarm provide containerization and cluster management, and the Traefik reverse proxy / load balancer (https://traefik.io/) manages requests and maintains sticky user sessions, which is essential in hosting Shiny applications for concurrent users. The scalable design makes it relatively easy to increase dedicated resources simply by adding more nodes to the Docker Swarm cluster, and thus to flexibly accommodate growth in computational demand as new applications are deployed and the user base expands. In addition, the platform has been deployed on AWS Cloud with Kubernetes (http://www.nasqar.com).

A Docker image of NASQAR is publicly available through DockerHub and can be used to deploy the application seamlessly on any system, whether a local computer or a public or private internet server (such as a research institute’s intranet). Although data uploaded online for analysis with NASQAR (at http://nasqar.abudhabi.nyu.edu/) is by default discarded after a user’s session ends, this does not guarantee total data privacy. Where privacy is a concern (e.g. patient data), NASQAR may be deployed on either a restricted intranet or a personal computer. Moreover, using Docker allows deployment of the entire NASQAR toolbox with a one-time install, removing the hassle of having to manually satisfy the different software requirements of numerous individual applications. The source code is publicly available on GitHub and is actively maintained. Each individual application is hosted in its own GitHub repository and can be accessed and launched independently via R or R Studio. All applications have clear user guides with example data sets to help users get started and acclimate quickly. This is a major factor in improving usability and thus adoption of the tools.

NASQAR’s collection of applications is primarily implemented in R, a widely used and freely available statistical programming language [18]. Most of the analysis workflows are built using R libraries for genomics and computation. The front-end design employs several R libraries, such as Shiny [19], shinydashboard, shinyjs, shinyBS, shinycssloaders. These libraries and custom Javascript/CSS/HTML enhancements improve the user experience and overall usability with interface consistency, visual clarity, and ease of navigation. Familiar R packages used to build the applications include dplyr and tidyr for matrix data manipulation; ggplot2, heatmaply, and NMF for figure plotting; and BiocParallel for multi-threading support. Additional packages used in conjunction with specific tools are indicated below.

In addition to previously published software, we introduce several new applications we have developed that wrap around popular analysis packages, such as DESeq2 [20] and Seurat [21, 22] for bulk and single-cell RNA-seq analysis and visualization. Since most NASQAR applications require a matrix of gene counts as input, we have also built a convenient tool to assist with pre-processing, GeneCountMerger. Some of the applications provide a seamless transition from data pre-processing to downstream analysis. This implementation gives users the option of using multiple analysis applications without having to modify/reformat the input data set, thus allowing them to easily benchmark and compare the performance of different analysis software packages.

## Results and discussion

NASQAR currently hosts tools for merging gene counts; conversion of gene IDs to gene names; and analysis of differential mRNA expression, gene function enrichment, and metagenomic profiling. Packages for bulk RNA-seq analysis include DESeq2, edgeR [23], and limma [24], while single-cell analysis is driven by Seurat (see Table 1 for a comprehensive overview of the applications). The Supplementary Materials include details on available applications along with example use cases. We believe the custom applications developed for NASQAR improve on several existing tools, as highlighted in the following application summaries.

**Table 1:** Comprehensive overview of NASQAR applications

	**Analysis type**	**Features / Functions**	**Main R Packages**	**Input**	**Output**	**Visualization**
**GeneCountMerger**	Preprocessing	Merge counts files into a matrix format that is needed for many downstream analysis; also offers gene id/name conversion	base R packages	gene count files (Eg. output count files from htseq)	csv file containing a matrix of samples and corresponding gene counts	N/A
**mergeFPKMs**	Preprocessing	Merge sample fpkm files into a matrix format; also offers gene id/name conversion	base R packages	sample gene fpkm files (Eg. sample fpkm files from Cufflinks)	csv file containing a matrix of samples and corresponding gene fpkm values	N/A
**Deseq2Shiny**	RNAseq (bulk)	Differential Gene Expression (DGE) analysis and optionally Surrogate Variable Analysis (SVA) for hidden batch effect detection	DESeq2, SVA	matrix of samples and gene counts (csv) and optionally a metadata table (csv)	VST matrix, Rlog matrix, DGE results table, Gene expression table, normalized counts matrix	Distance heatmap, PCA plots, MA plots, Gene expression boxplots, normalized counts heatmap, SVA plots
**START**	RNAseq (bulk)	Differential Gene Expression (DGE) analysis	edgeR, limma-voom	matrix of samples and gene counts (csv)	Gene expression table	PCA plots, Heatmaps, Scatter plots, Volcano plots, Gene expression boxplots
**DEApp**	RNAseq (bulk)	Differential Gene Expression (DGE) analysis	DESeq2, edgeR, limma-voom	matrix of samples and gene counts (csv) and a metadata table (csv)	DGE results table, method comparison results	PCA plots, Dispersion plot, Volcano plot, Venn diagram method comparison
**Seurat(V3)Wizard**	RNAseq (single-cell)	Guided single-cell RNA-seq data analysis and clustering.	Seurat, sctransform, dplyr	either 10X data files (mtx, tsv) OR matrix of cell/gene counts	PCA results, ICA results, DGE cluster markers table, Seurat R Object, R script	Violin plots, PCA plots, PCA heatmaps, Elbow Plot,UMAP, t-SNE
**Shaman**	Metagonomics	Differential analysis of quantitative metagenomic data	DESeq2, circlize, ape, phytools, philentropy	a BIOM matrix file OR a counts file (csv)/taxonomy file (tsv). Optionally a phylogenetic tree newick file (nhx) and/or fastq files	Output files (biom, tsv, nhx) of Shaman workflow that can be used in downstream metagenomic analysis and visualization within Shaman	Bar plots, PCOA/PCA plots, Clustering dendograma, Rarefaction curves, Scatter plots, Heatmaps, Box plots, Diversity plots, Venn diagram
**ClusterProfShinyGSEA**	Gene Enrichment - Gene Set Enrichment Analysis	Gene Set Enrichment Analysis (GSEA) of GO-Terms and KEGG pathways	clusterProfiler, DOSE, Goplot, enrichplot, pathview	a table of differential gene expression DGE data (csv/tsv)	GO terms table, KEGG results table	Dot plots, Category netplot, GO induced graph, Pathview plot, ridge plot, pubmed trends
**ClusterProfShinyORA**	Gene Enrichment - Over Respresentation Analysis	Over-representation analysis of GO-Terms and KEGG pathways	clusterProfiler, DOSE, Goplot, enrichplot, pathview, wordcloud2	a table of differential gene expression DGE data (csv/tsv)	GO terms table, KEGG results table	Bar plots, Dot plots, Category netplot, GO induced graph, Pathview plot, word cloud

### *GeneCountMerger*

**Fig. 2:**
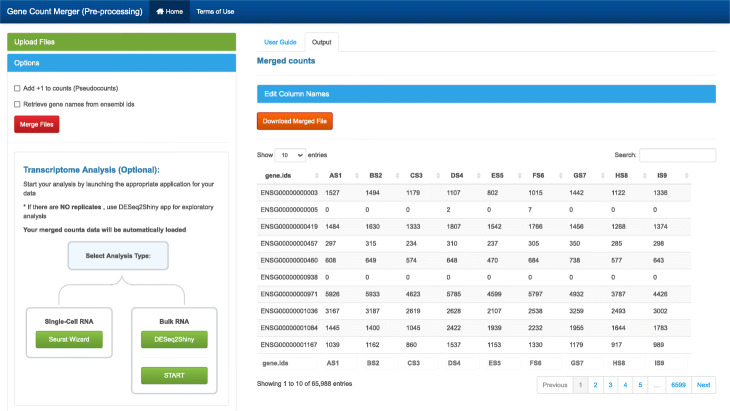
GeneCountMerger screenshot. A preprocessing utility to generate the gene count matrices required as input to many analysis tools. It can merge individual raw gene count files from htseq-count and other similar applications. Convenient features include conversion of Ensembl gene IDs to gene names for reference genomes and seamless launching of downstream analysis applications
- Merge multiple raw count matrices
- Convert Ensembl gene IDs to gene names
- Select from available genomes / versions
- Add pseudocounts
- Rename sample column headers
- Download merged counts file in.csv format
- Seamless transcriptome analysis following merging counts (Seurat Wizard for single-cell RNA analysis; DESeq2Shiny or START [10] for bulk RNA analysis)

This preprocessing tool is used to merge individual raw gene count files produced from software such as htseq-count [25] or featurecounts [26] (Fig. 2). Options include:

- Merge individual sample count files into one matrix

### *Seurat wizards*

**Fig. 3:**
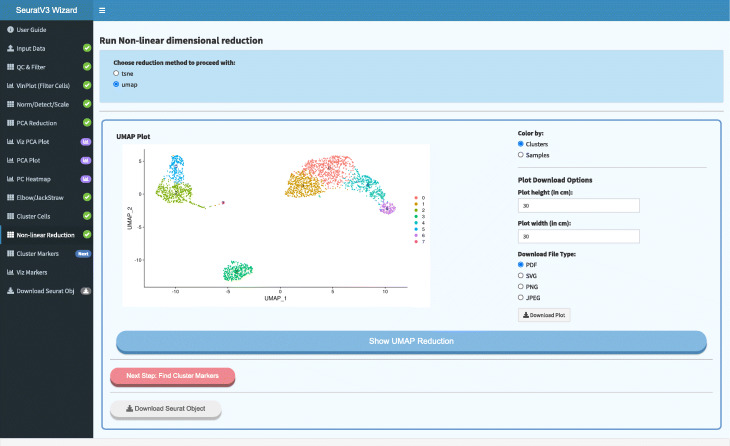
Seurat wizard screenshot. Wizard-style web-based interactive applications based on Seurat, a popular R package designed for QC, analysis, and exploration of single-cell RNA-seq data. The wizards guide users through single-cell RNA-seq data analysis and visualization and provide an intuitive way to fine-tune parameters using feedback from results at each stage of the analysis. Functionality includes filtering, normalization, dimensionality reduction (PCA), clustering, and visualization with UMAP or t-SNE plots

Seurat Wizards are wizard-style web-based interactive applications to perform guided single-cell RNA-seq data analysis and visualization using Seurat, a popular R package designed for QC, analysis, and exploration of single-cell RNAseq data (Fig. 3). The design and implementation of the wizards offer an intuitive way to tune the analysis interactively by allowing users to inspect and visualize the output of intermediate steps and adjust parameters accordinly. In contrast, most web-based tools for scRNA-seq analysis, such as IS-CellR [14] and SCHNAPPs (https://c3bi-pasteur-fr.github.io/UTechSCB-SCHNAPPs/), provide integrated solutions that offer less opportunity for user intervention at intermediate steps. Some of the distinctive features of the wizards include, 1) allowing users to visually inspect the distribution of cells using violin plots and to select cutoff thresholds accordingly in order to filter out cells before starting the analysis, 2) Elbow/Jackstraw plots that assist the users in determining what dimensions to use for non-linear reduction. Both of these features can have significant consequences on downstream steps like clustering and differential analysis.

The Seurat Wizards follow closely the Seurat Guided Clustering Tutorials devised by the Seurat authors (https://satijalab.org/seurat/v3.0/pbmc3k_tutorial.html). Both Seurat versions 2 and 3 are currently supported. Users can follow the tutorials while using the Wizards and edit parameters at almost every step, which is instrumental in producing accurate results. Pre-processing (QC/filtering), normalization, dimensionality reduction, clustering (UMAP/t-SNE), and differential expression (cluster biomarkers) are all supported. To enhance the user experience and learning process, the wizards progress step-wise through the workflow. The workflow processing steps become available sequentially upon completion of each preceding task, thus avoiding visual clutter and focusing the user’s attention on the task at hand. One of the unique features of the Seurat Wizards is that they can accept as input either processed 10X Genomics data files or a matrix of gene counts, which eliminates the need for an additional pre-processing step. To address reproducibility, the last step of the wizard allows the user to download an R script with all of the R functions and parameters used for the analysis, along with the R object that contains all of the analyzed data, for further exploration in R/RStudio.

SeuratV3Wizard integrates several additional features like the UCSC Cell Browser (https://github.com/maximilianh/cellBrowser), which enables users to interactively visualize clusters and gene markers. Additional cell browser options will be implemented in future releases where feasible. It also includes the newly published sctransform method [27], which offers users the convenience of running an analysis using two slightly different workflows and comparing the results. We believe these differences in features and design give the Seurat Wizards more versatility and improve usability in comparison with other publicly available implementations.

### *DESeq2Shiny*

**Fig. 4:**
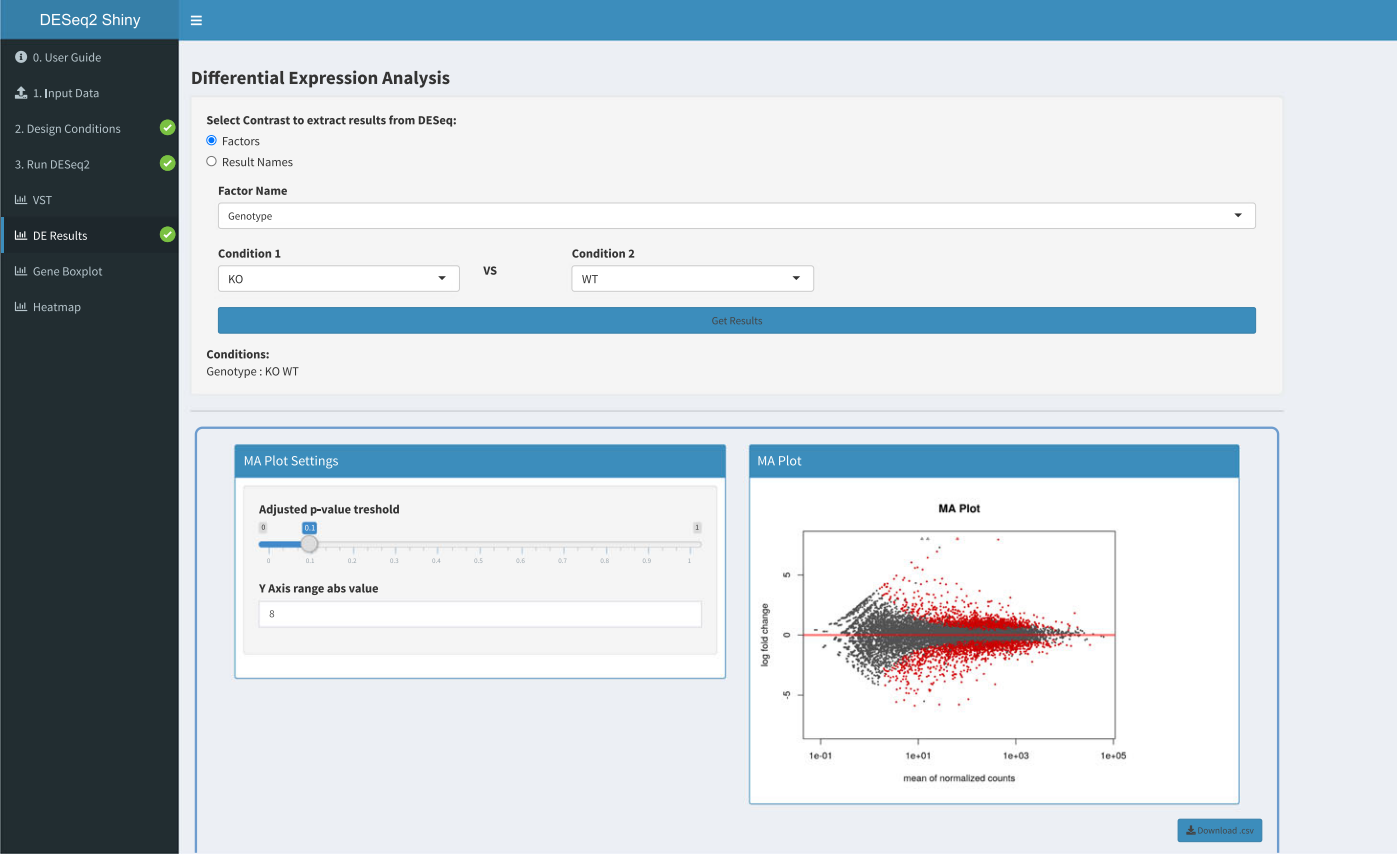
DESeq2Shiny screenshot. A web-based shiny wrapper around DESeq2, a popular R package for performing differential mRNA expression analysis of RNA-seq data

The DESeq2Shiny app is a Shiny wrapper around DESeq2, a popular R package for performing differential mRNA expression analysis of RNA-seq data (Fig. 4). This web-based application provides functions for data normalization, transformation (e.g., rlog and vst for clustering), and estimation of dispersion and log fold-change. The results are all downloadable in csv format. Data visualizations include MA plots, heatmaps, dendograms, gene expression boxplots, and PCA.

The application is capable of working with simple experimental designs or complex experiments with multiple factors. For single-factor experiments with replicates, sample names can be parsed and grouped automatically given proper formatting. The experimental design table and formula can also be generated autonomously. For multifactor experiments, the table can be constructed easily within the "Edit Conditions" page, or an experiment design metadata (csv) file may be uploaded directly. The design formula expresses how the counts for each gene depend on the factor(s) and is editable within the “Edit Conditions” page. This gives users the option to specify experimental designs with multiple variables (e.g. *~group*+*condition*) and interaction terms (e.g. *~genotype*+*treatment*+*genotype*:*treatment*). In cases where no replicates exist, exploratory analysis (with no differential testing) may be performed by setting the formula to *~*1 (which signifies “no design”). Most other surveyed R Shiny applications for RNAseq differential gene expression (DGE) analysis (e.g. START, DEApp, TCC-GUI, and Shiny-seq) lack such flexible features. While users are warned that this type of analysis has low statistical power and they should not draw strong conclusions based on the results, it is none the less implemented as a feature to allow for exploratory testing and hypothesis generation [28].

The DESeq2Shiny app interface design follows the same implementation as other apps on NASQAR: users are guided through the analysis, and subsequent steps become available when the current step is completed and valid. Users may also fine-tune analysis parameters interactively. This design, coupled with preloaded example datasets for single or multi-factor designs, aims to improve ease of use. Known batch effects can be modeled simply by adding the batch as a factor in the design matrix and formula. The application also offers hidden batch effect estimation using svaseq [29]. This allows for the estimation of surrogate variables, which can be included as adjustment factors in the design formula to correct subsequent downstream analysis.

### *ClusterProfShiny*

**Fig. 5:**
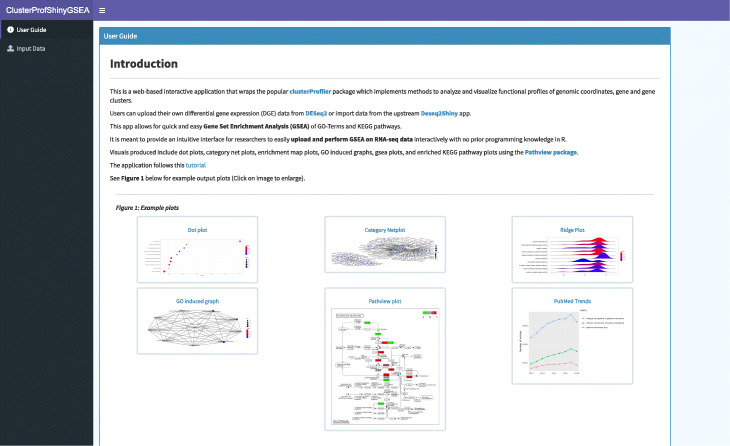
ClusterProfShinyGSEA screenshot. Web-based apps wrap the popular R package clusterProfiler for the analysis and visualization of functional themes and enrichment among gene clusters, using data from either DESeq2 or DESeq2Shiny. Both Gene Set Enrichment Analysis (GSEA) and Over-Representation Analysis (ORA) are implemented

The ClusterProfilerShiny apps wrap the popular clusterProfiler [30] package, which implements methods to analyze and visualize functional profiles of genomic coordinates, genes, and gene clusters (Fig. 5). Users can upload their own data from the output of DESeq2, for example, or import analyzed data from the upstream DESeq2Shiny app. These apps allow for quick and easy over-representation analysis (ORA) and gene set enrichment analysis (GSEA) of GO terms and KEGG pathways. Visuals produced include dot plots, word clouds, category net plots, enrichment map plots, GO induced graphs, GSEA plots, and enriched KEGG pathway plots using the Pathview [31] package.

### Other open-source apps

- **START**: a web-based RNA-seq analysis and visualization resource using edgeR and limma-voom. We have modified this application slightly from the published version to add options to some plots. We have also integrated it with GeneCountMerger so that once merging gene counts is complete, users may launch the START app and have their merged matrix data loaded automatically.
- **DEApp** [11]: an interactive web application for differential expression analysis using DESeq2, edgeR, limma-voom.
- **Shaman** [15]: a Shiny application that enables the identification of differentially abundant genera within metagenomic datasets. It wraps around the Generalized Linear Model implemented in DESeq2. It includes multiple visualizations and is compatible with common metagenomic file formats.

### Ongoing and future work

Numerous feature enhancements are planned or in progress to improve and expand functionality. For example, SeuratV3Wizard now provides the option to download an auto-generated R script and R object containing the executed code and results of a Seurat analysis. While all NASQAR applications currently expose functions/workflow parameters in the UI (so they are visible to the user), and analysis results are easily downloadable, having the ability to download the entire workflow in R enhances reproducibility by allowing users to document and share the specific commands and parameters used. Adding this option for other applications such as DESeq2shiny and ClusterProfiler(ORA/GSEA) will help users learn to understand their workflows in greater depth and will foster collaboration between experimental and computational biologists. In addition, we are continuously working to provide more online documentation for different use cases, to improve error handling for all NASQAR apps, and to evaluate possibilities for additional domain applications such as single-molecule long-read data. In order to facilitate broader deployment and ease of access, we have deployed NASQAR on AWS Cloud (available at www.nasqar.com). Cloud computing services open up opportunities for new analysis and visualization categories such as variant discovery, which requires both heavy computation and a large amount of data storage.

## Conclusion

The NASQAR platform offers a publicly available, comprehensive toolbox of interactive bioinformatics and visualization applications for sequence analysis that is accessible to all researchers with or without computational experience. NASQAR online services are currently deployed through NYU (with Docker/Swarm) and on AWS Cloud (with Kubernetes). In cases where data privacy is a major concern, the entire platform can be deployed privately on a personal computer or as a shared resource on a local intranet. Google Analytics traffic reports and GitHub activity show that the global user base is diverse and is increasing steadily, revealing rising demand in the community for easily accessible bioinformatics analysis and visualization platforms. NASQAR is under active development and will continue to offer user support and feature enhancements with future releases.

## Supplementary information

**Additional file 1** This file includes supplementary materials such as instructions on how to launch NASQAR and example use cases on data analysis and visualization.

## Data Availability

NASQAR is publicly accessible at http://nasqar.abudhabi.nyu.edu/ and http://www.nasqar.com/. The platform is available as a Docker image at https://hub.docker.com/r/aymanm/nasqarall. NASQAR is open-source and the code is available through GitHub: NASQAR (main page): https://github.com/nasqar/NASQAR SeuratV3Wizard (scRNA): https://github.com/nasqar/seuratv3wizard SeuratWizard (scRNA): https://github.com/nasqar/SeuratWizard deseq2shiny (Bulk RNA): https://github.com/nasqar/deseq2shiny GeneCountMerger (Pre-processing): https://github.com/nasqar/GeneCountMerger ClusterProfShinyGSEA (Enrichment): https://github.com/nasqar/ClusterProfShinyGSEA ClusterProfShinyORA (Enrichment): https://github.com/nasqar/ClusterProfShinyORA

## Abbreviations

NASQAR: Nucleic acid SeQuence analysis resource
RNA-seq: RNA sequencing
NGS: Next generation sequencing
CRISPR: Clustered regularly interspaced short palindromic repeats
GUI: Graphical user interface
PCA: Principal components analysis
t-SNE: t-Distributed stochastic neighbor embedding
UMAP: Uniform manifold approximation and projection
QC: Quality control
UCSC: University of California, Santa Cruz
rlog: Regularized logarithm transformation
vst: Variance stabilizing transformation
ORA: Over-representation analysis
GSEA: Gene set enrichment analysis
GO: Gene ontology
KEGG: Kyoto encyclopedia of genes and genomes
START: Shiny transcriptome analysis resource tool
Shaman: SHiny application for Metagenomic ANalysis
csv: Comma separated values
